# Effect of short-term 10 Hz repeated transcranial magnetic stimulation on postural control ability in patients with mild hemiparesis in acute ischemic stroke: a single-blinded randomized controlled trial

**DOI:** 10.3389/fneur.2024.1439904

**Published:** 2024-08-14

**Authors:** Jiangping Ma, Siyu Qian, Nuo Ma, Lu Zhang, Linghao Xu, Xueyuan Liu, Guilin Meng

**Affiliations:** ^1^Department of Neurology, Shanghai Tenth People’s Hospital, Tongji University School of Medicine, Shanghai, China; ^2^Department of Neurology, Tongren Hospital, Shanghai Jiao Tong University School of Medicine, Shanghai, China; ^3^Department of Neurology, Qingpu Branch of Zhongshan Hospital, Fudan University, Shanghai, China; ^4^Department of Cardiology, Shanghai East Hospital, Tongji University School of Medicine, Shanghai, China

**Keywords:** ischemic stroke, repetitive transcranial magnetic stimulation, rehabilitation, postural control, gait

## Abstract

**Background:**

Previous studies have demonstrated that repetitive transcranial magnetic stimulation (rTMS) can improve postural control in subacute and chronic ischemic stroke, but further research is needed to investigate the effect of rTMS on acute ischemic stroke.

**Objective:**

We compared the therapeutic effects of rTMS plus conventional rehabilitation and conventional rehabilitation on postural control in patients with mild hemiparesis in acute ischemic stroke.

**Methods:**

Eighty-six patients with acute ischemic stroke were randomly assigned to either the experimental group or the control group within 1–7 days of onset. Patients in both groups received conventional rehabilitation for 2 weeks. Patients in the experimental group received rTMS treatments lasting for 2 weeks. Before and after the 2-week treatment, patients were assessed based on the Timed up and Go (TUG) test, Dual-Task Walking (DTW) test, Functional Ambulation Category (FAC), Tinetti Performance Oriented Mobility Assessment (POMA), gait kinematic parameters, Barthel Index (BI), Mini-Mental State Examination (MMSE), Montreal Cognitive Assessment (MoCA), and National Institutes of Health Stroke Scale (NIHSS). Additionally, TUG and single-task gait velocity were assessed at 2 months after the start of treatment, and independent walking recovery was also followed up.

**Results:**

After 2 weeks of treatment, compared to conventional rehabilitation, participants who underwent rTMS treatment plus conventional rehabilitation exhibited notable enhancements in TUG, FAC, POMA, and some gait parameters [single-task gait velocity, gait stride length, gait cadence, gait cycle]. Changes in cognitive function partially mediated the improvement in single-task gait velocity and gait stride length by rTMS plus conventional rehabilitation. Generalized Estimating Equation (GEE) analysis showed that the trend of improvement in single-task gait velocity over time was more pronounced in the experimental group than in the control group. The results of the Kaplan–Meier curve indicated a median gait recovery time of 90 days for patients in the experimental group and 100 days for the control group. Multifactorial Cox regression analyses showed that rTMS plus conventional rehabilitation promoted faster recovery of independent walking compared with conventional rehabilitation.

**Conclusion:**

rTMS plus conventional rehabilitation outperformed conventional rehabilitation in improving postural control in patients with acute ischemic stroke. Improvements in cognitive function may serve as a mediating factor in the favorable treatment outcome of rTMS plus conventional rehabilitation for improving postural control.

**Clinical trial registration:**

https://www.chictr.org.cn, identifier ChiCTR1900026225.

## Introduction

Stroke is an acute focal injury to the central nervous system of vascular origin, causing neurological deficits (1). Ischemic stroke, which carries a lifetime risk of 18.3% worldwide, is the third most common cause of adult disability (2). Postural control (PC) refers to the body’s ability to maintain stability and orientation through the motor system by integrating information from somatosensory, vestibular, and visual inputs (3–5). After a stroke, various abnormalities such as reduced muscle strength, impaired feedforward mechanisms, sensory deficits, and cognitive impairment can arise, leading to a reduction in postural control (6–10). Postural control is significantly associated with decreased mobility and impaired ability to carry out daily activities, and it is one of the main risk factors for falls in stroke patients (11). Relevant studies have demonstrated that stroke patients have a high incidence rate of falls within 6 months, ranging from 37 to 73% (12–14). Therefore, rehabilitation programs should prioritize enhancing postural control to prevent falls.

In clinical practice, traditional conventional manipulative rehabilitation is considered relatively effective for restoring neurological function in stroke patients, but its effectiveness is constrained (15, 16). In recent years, numerous novel rehabilitation therapies have emerged, including virtual reality (VR) technology, repetitive transcranial magnetic stimulation (rTMS), dual-task training (DT), and others (17–19). rTMS is a non-invasive neuromodulation therapy that can modify the excitability of the cerebral cortex and restore the inhibitory balance of both hemispheres (20, 21), resulting in noteworthy enhancement in neurological function of individuals who experienced a stroke (22). The impact of rTMS on the functional rehabilitation of stroke patients’ upper limbs in the acute phase is relatively evident (23). However, further research is needed to provide additional evidence for the therapeutic effectiveness of rTMS in restoring motor function in the lower limbs of patients in the acute stage of stroke (24). A recent systematic review and meta-analysis suggests that stimulating the primary motor cortex (M1) area with rTMS significantly improves walking speed, balance, and postural control in stroke patients (25–27). However, Huang and colleagues discovered that applying 1 Hz low-frequency rTMS (LF-rTMS) to the cortex opposite the lesion did not result in a significant improvement in motor or walking capabilities for stroke patients (28). The therapeutic effect of rTMS on lower limb motor function in stroke patients still remains controversial. Regarding the optimal timing of lower limb motor rehabilitation in stroke patients, clinical evidence only supports the use of rTMS in the subacute phase of stroke (1–6 months after stroke) and in the chronic phase of stroke (> 6 months after stroke) to improve balance and gait, and more evidence-based medical evidence is needed for the use of rTMS in the acute phase of stroke (< 1 month after stroke onset) (29, 30). Hence, further research is necessary to generate more advantageous clinical evidence supporting the utilization of rTMS in the rehabilitation of lower limbs in stroke patients and to continue advancing the field of rTMS.

Although there is evidence of the efficacy of rTMS in lower limb motor rehabilitation of stroke patients, experimental studies have used different stimulation times, intensities, and sites. Furthermore, stroke patients are at varying stages of the disease, resulting in significant outcome variability. The efficacy of some treatments remains contentious, and concise and standardized treatment protocols backed by evidence have yet to be established. The absence of high-quality clinical studies with large samples hinders the formulation of a consensus on rTMS treatment guidelines, thus impeding the widespread use of rTMS in lower limb motor rehabilitation for stroke patients. The aim of this study is to investigate the clinical effectiveness of transcranial magnetic stimulation plus conventional rehabilitation in lower limb motor rehabilitation for patients with mild hemiparesis in acute ischemic stroke and provide a foundation for post-stroke rehabilitation strategies for postural control disorders.

## Materials and methods

### Ethical considerations and study design

The study procedure was reviewed and approved by the Ethics Committee of Shanghai Tenth People’s Hospital (SHSY-IEC-BG/05.08/05.0) and was registered in the Chinese Clinical Trial Registry (no. ChiCTR1900026225). All participating patients were duly informed of the study procedures, its safety, and signed an informed consent form. The study was conducted in compliance with the Declaration of Helsinki principles. It was a prospective single-blind randomized controlled trial conducted at a single center. The trial was divided into experimental and control groups according to treatment measures, with an equal number of patients in each group. Participants were randomly assigned to either group. Data were collected from May 28th, 2023 to October 15th, 2023.

### Participants

**Figure 1:**
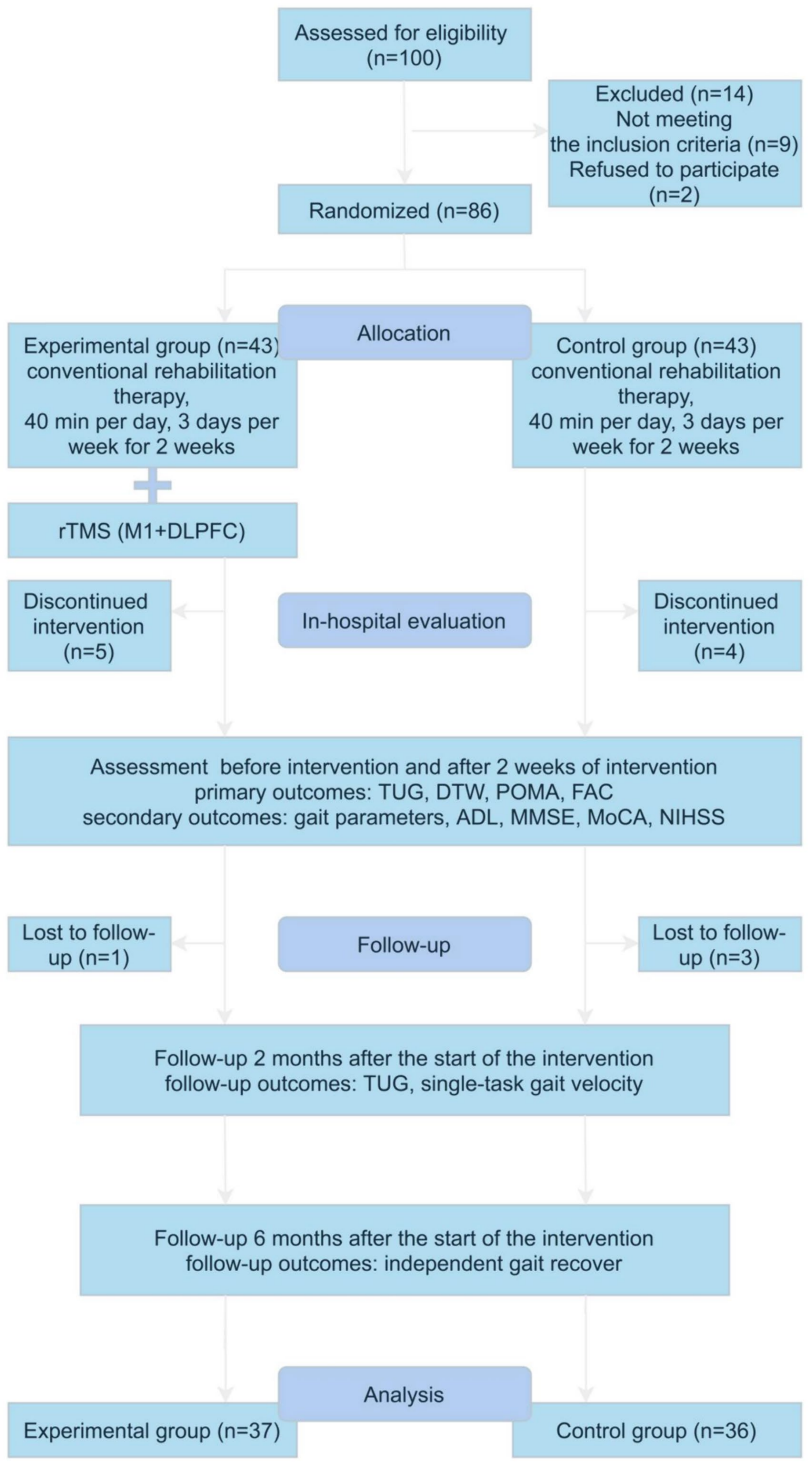
Flow diagram of the study. Gait parameters including single-task and dual-task gait velocity, stride length, cadence, and cycle durations. rTMS, repetitive transcranial magnetic stimulation; M1, primary motor cortex; DLPFC, left dorsolateral prefrontal cortex; TUG, time up and go test; DTW, dual-task walking test; POMA, tinetti performance oriented mobility assessment; FAC, functional ambulation classification; ADL, activities of daily living; MMSE, mini-nental state examination; MoCA, montreal cognitive assessment; NIHSS, National Institute of Health Stroke Scale.

Eighty-six patients with acute ischemic stroke who were hospitalized at the Department of Neurology, Shanghai Tenth People’s Hospital from May 28, 2023 to September 10, 2023, participated in this study (The initial screening of 100 patients revealed that 9 did not meet the inclusion criteria and 2 declined to participate in the study) (Figure 1). All participants fulfilled the following criteria: (1) patients who met the diagnostic criteria for acute anterior circulation ischemic stroke according to the Chinese Guidelines for the Diagnosis and Treatment of Acute Ischemic Stroke (2018); (2) adults aged 18 to 80 years; (3) patients with first onset of disease, presence of hemiparesis, and lower limb muscle strength of grade IV or higher; (4) patients with ability to stand unaided for at least 5 min; (5) patients with Mini-Mental State Examination (MMSE) score of over 17 points and ability to cooperate in completing the cognitive and gait assessment; and (6) patients who had signed informed consent. Patients with any of the following conditions were excluded: (1) unstable vital signs; (2) severe cardiac, pulmonary, renal, hepatic, or other organ disease; (3) limb dysfunction prior to brain lesions; (4) other causes of postural control abnormalities such as vestibular system disorders, visual and auditory abnormalities, peripheral nerve, and musculoskeletal pathologies; (5) severe cognitive deficits, major depression, and inability to cooperate with cognitive and balance assessments; (6) presence of metallic implants such as pacemakers or cochlear implants; and (7) epilepsy or use of medications that affect cortical excitability.

The dropout criteria for this trial were as follows: (1) subjects who voluntarily discontinued during the trial; (2) patients who changed their treatment regimen during the trial; and (3) serious side effects or complications. The trial should be stopped if any of the following occurs: (1) serious adverse reactions, special physiological changes, or other unexpected events occur during the trial that make it difficult to continue participation in the trial; (2) patients experience serious complications or deterioration in their condition during the trial and require emergency treatment; (3) subjects withdraw from the trial midway through the trial; and (4) subjects fail to cooperate or comply with treatment after repeated explanations by clinicians.

After applying the inclusion and exclusion criteria, 86 participants were selected and randomized to the experimental group and control group. However, nine participants withdrew from the study within 2 weeks of the intervention and four participants were lost to follow-up at a later date. Thus, the final study population consisted of 73 subjects, 37 in the experimental group and 36 in the control group.

Prior to the intervention, we collected basic clinical information, rehabilitation assessment data, postural control disorder scale, cognitive assessment, activities of daily living ability, gait video, and other relevant data for the enrolled patients. After the intervention, we evaluated the postural control disorder scale, cognition, ability to perform daily activities, gait, and other data. All results were assessed by blinded assessors. The researchers knew which group the patients were allocated to, but the patients, outcome assessors, and data analysts did not. The data were collected at the Neurological Rehabilitation Center, Department of Neurology, Shanghai Tenth People’s Hospital.

### Grouping and interventions

A randomized study design was used in this trial. For patients who met the enrollment criteria, the R project was used to generate a random allocation table and they were randomly assigned to the experimental group and control group in a 1:1 ratio. The principal investigator used the R project to generate the random allocation sequence for study participants, and then the random allocation sequence was concealed in sequentially numbered sealed opaque envelopes, which were opened sequentially by each patient after signing the informed consent form. Patients in the control group received conventional rehabilitation training, including head, neck, and trunk control training, dynamic sitting balance training, dynamic standing center of gravity shift training, dynamic standing balance training (hip, ankle, stride strategy), and walking training. The conventional rehabilitation training was conducted for 40 min per session, three times a week, for 2 weeks. Patients in the experimental group received 10 Hz high-frequency TMS stimulation before conventional rehabilitation training three times a week for 2 weeks. The stimulation sites were the primary motor cortex (M1) and left dorsolateral prefrontal cortex (DLPFC) areas. The resting motion threshold (rMT) was defined as the lowest stimulus intensity that elicited minimal ankle motion on at least five out of ten consecutive training sessions (Our rTMS was targeted at the hand representation area, not the leg. Stimulation over C3/C4 with high enough intensity could cause activation of the leg area, producing the ankle movement we used to determine rMT). The M1 area was stimulated at a frequency of 10 Hz, with an intensity of 90% rMT and the treatment protocol involved a 50-s pause between each 10-s pulse (100 pulses in total), repeated 10 times, resulting in a total of 1,000 pulses per session. The DLPFC area was stimulated at a frequency of 10 Hz, with an intensity of 80% rMT and the treatment protocol involved a 25-s pause between each 5-s pulse (50 pulses in total), repeated 40 times, resulting in a total of 2,000 pulses per session. Patients in experimental group received rTMS to both (M1 and DLPFC) targets during each rTMS session. The patient wore a 10–20 system EEG cap for scalp positioning and earplugs for hearing protection (The left M1 region was localised at lead C3 and the right M1 region was localised at lead C4, while fine adjustments were made in conjunction with rMT measurements. The left DLPFC region was positioned at lead F3). The patients received rehabilitation from a nationally accredited therapist with over 5 years of experience. The assignment of patients to groups was only known by the investigators of the trial, and not by the patients, outcome assessors, or data analysts.

### Outcome measures

**Figure 2:**
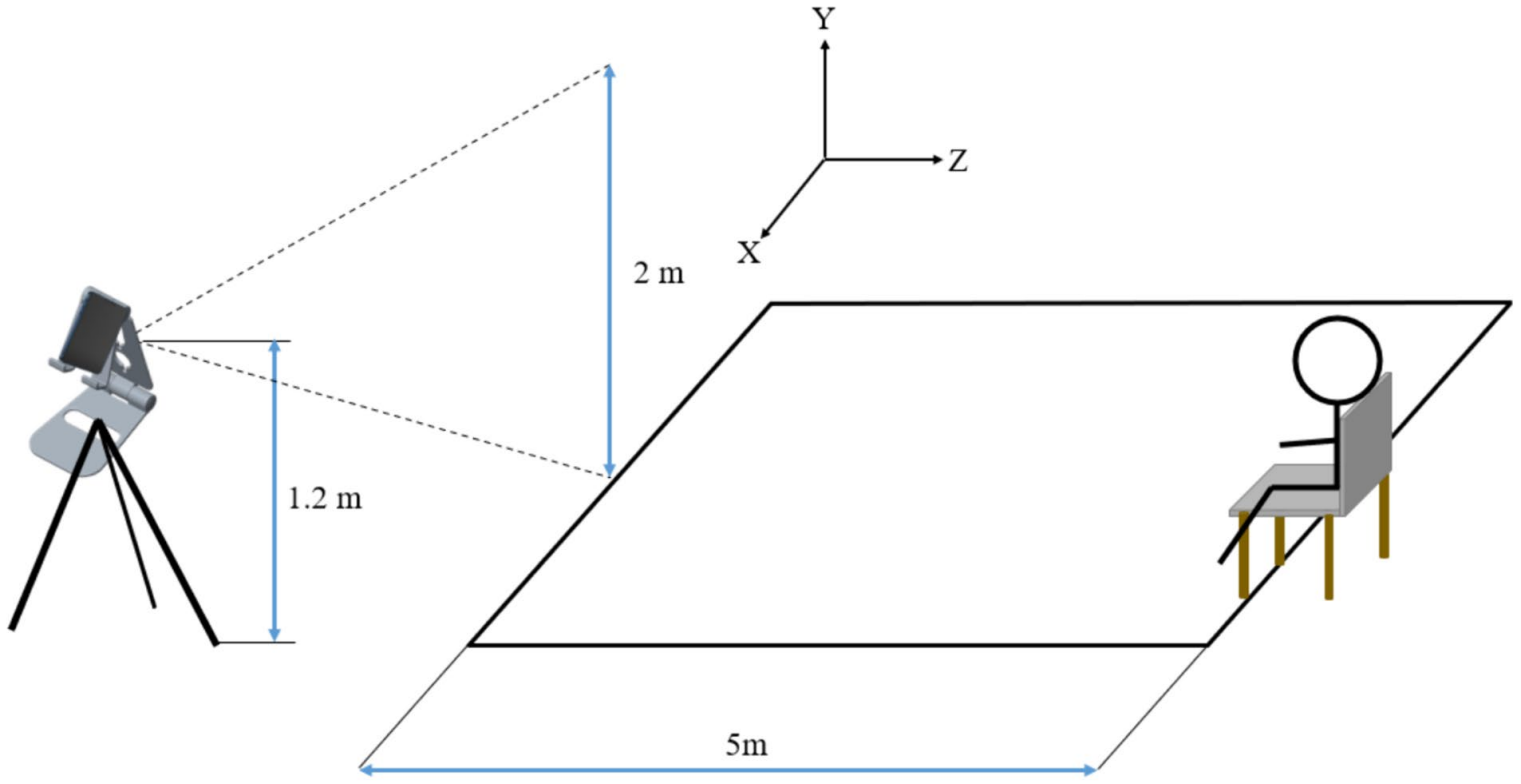
Video gait analysis.

The primary outcome measures were the Timed up and Go (TUG) test, the Dual-Task Walking (DTW) test, the Functional Ambulation Category (FAC), and the Tinetti Performance Oriented Mobility Assessment (POMA). Secondary outcome measures were gait kinematic parameters, activities of daily living (ADL), Mini-Mental State Examination (MMSE), Montreal Cognitive Assessment (MoCA), and National Institutes of Health Stroke Scale (NIHSS). During the intervention, patients were assessed before the intervention and 2 weeks after the start of the intervention. Long-term follow-up outcomes included patients’ TUG and single-task gait velocity 2 months after treatment, time to return to completely independent walking (completely independent walking is defined as a 5-score FAC and the participant’s subjective absence of any sensation of hemiparesis). The TUG is a rapid screening tool used to assess daily mobility and balance problems in older adults (31). The test requires the patient to stand up from a chair, walk 3 meters forward, turn around, and then return to sit down. The patient’s postural control is scored based on the time taken, with longer times indicating poorer postural control (31). In this study, a 5-meter distance was used to test the patients’ TUG in order to detect potential differences in pre- and post-intervention changes in postural control between different groups. The DTW was used to measure the patients’ postural control under the dual task of motor and cognitive performance by having the patients perform a TUG in conjunction with a 100-7 sequential calculation (32). The FAC was used to assess the patients’ walking ability and was scored according to the degree to which the patient relied on external support to walk, with scores ranging from 0–5, and the higher the score, the better the walking ability (33). The POMA was used to assess the patient’s gait and balance, with scores ranging from 0–28, and the higher the score, the better the patient’s postural control (34). Gait kinematic parameters were analyzed from the videos recording the patients during TUG and DTW to obtain the patients’ gait velocity, gait stride length, gait cadence, and gait cycle in single and dual tasks (Figure 2). The BlazePose architecture was employed for the purpose of gait analysis. BlazePose represents a lightweight convolutional neural network architecture that has been specifically designed for the estimation of human poses on mobile devices (35). In the initial stage, the BlazePose model infered 33 2D keypoints of the human body, which accurately described the human body’s pose, from a single frame image. Subsequently, the coordinate data pertaining to these keypoints in successive frames were collated to construct a time series. Subsequently, the aforementioned time series data were employed for the purpose of gait analysis. By comparing and analysing the coordinate changes of keypoints in adjacent frames, it was possible to extract a number of gait-related parameters, including step count, gait stride length, gait gadence and gait velocity. The number of steps were estimated by detecting the periodic changes in the coordinates of the keypoints, with each periodic change in the keypoints representing one step. The distance between two consecutive key points, where the heel of the same side lands, was calculated to determine the gait stride length. Gait cadence was determined by calculating the number of steps completed in a given time period, typically expressed in steps per second. Gait velocity was calculated by dividing the product of the gait stride length and gait cadence by 2. ADL was assessed by using the modified Barthel Index (BI) with a score range of 0–100 (36). MMSE and MoCA were used to assess the patients’ overall cognitive function (37, 38). NIHSS was used to assess the degree of neurological deficit in patients (39). At each follow-up time point, two experienced neurologists conducted an independent assessment of the patients’ primary outcome indicators and secondary outcome indicators, respectively. The individual responsible for administering the transcranial magnetic stimulation was not involved in the clinical assessment process, and the rehabilitation physician was unaware of the patient subgroups. The outcome assessors were blinded to the grouping and interpretation of the data.

### Sample size

Referring to the previous study of rTMS in postural control ability in stroke (40), combined with the inclusion criteria of this study, the primary endpoint indicator (TUG), the test efficacy 1−β = 90%, the test level α = 0.05, the expected mean of 29.96 in the experimental group with a standard deviation of 4.28, the expected mean of 30.56 in the control group with a standard deviation of 2.72, and the 1:1 ratio of the number of people included in the experimental group to the control group, according to the sample size calculation formula for comparing the means of the two samples, it was calculated that 24 cases were needed in each of the two groups, and considering a failure rate of 20%, a total of at least 60 cases needed to be included.

### Statistical analysis

SPSS 25.0 (version 25.0; IBM Corp., Armonk, NY, United States) and R 4.2.2 were used for statistical analysis. The Shapiro–Wilk test was used to test the normality of continuous variables. Continuous variables that conformed to a normal distribution were presented as mean ± standard deviation, and the *t*-test was used for comparison. Continuous variables not conforming to the normal distribution are presented as median (interquartile range) and the Mann–Whitney U test was used for comparison. Categorical variables were expressed as frequencies and rates, and comparisons were made by using the χ2 test (bicategorical variables) or the Wilcoxon rank-sum test (ranked variables). For continuous outcome variables, the Mann–Whitney U test was used to analyze change scores to analyze differences between comparison groups. The change value was defined as the improvement in the measured data 2 weeks after the start of the intervention compared with the baseline data before the intervention.

The repeated measures (TUG and single-task gait velocity) were analyzed using generalized estimating equations (GEE). The independent variables were rehabilitation treatment measures, measurement time, and the interaction between rehabilitation treatment measures and measurement time.

To analyze the mediating role of cognitive function in the improvement of postural control with rTMS plus conventional rehabilitation, mediation analysis was conducted, and the ratio of indirect effects to total effects was used to quantify the effect size of the mediation model.

Survival analysis (log-rank test) was used to compare the long-term effects of the different interventions on the outcome events (independent walking recovery). Multivariate Cox regression analysis was used to identify predictors of independent walking recovery.

The criterion for statistical significance was set at *p* < 0.05.

## Results

### Patients’ general characteristics

A total of 73 patients completed the entire study protocol, with 9 participants withdrawing from the study within 2 weeks of the intervention and 4 participants subsequently lost to follow-up, as shown in the graph (Figure 1). The baseline characteristics of the patients are shown in Table 1. All patients were well compliant with treatment and experienced no adverse events.

**Table 1:** Baseline data comparison.

Variable	Control group (*n* = 36)	Experimental group (*n* = 37)	*p* value
Age(years)	65.83 ± 8.45	68.62 ± 10.67	0.221
Male, *n* (%)	25 (69.44)	23 (62.16)	0.512
Height (cm)	165.75 ± 8.12	164.86 ± 8.01	0.641
Weight (kg)	67.08 ± 11.62	63.60 ± 11.99	0.212
NHISS	2.00 ± 1.41	2.54 ± 1.35	0.100
Brunnstrom stage	5.03 ± 056	4.78 ± 0.58	0.073
Side of lesion			0.935
Left, *n* (%)	19 (52.78)	21 (56.76)	
Right, *n* (%)	11 (30.56)	10 (27.03)	
Both, *n* (%)	6 (16.66)	6 (16.21)	

NIHSS, National Institute of Health Stroke Scale. *p* value was calculated according to the *T*-test or Chi-square test.

### Treatment effects

The rating scales of the experimental and control groups at baseline and after 2 weeks of treatment are shown in Table 2. Compliance was satisfactory and all patients completed all treatments.

**Table 2:** Comparison of scale assessment between two groups.

Variable		Control group (*n* = 36)			Experimental group (*n* = 37)			*p*
		T0	T1	Difference	T0	T1	Difference	
TUG	TUGT(s)	24.85 (10.90)	23.24 (9.17)	1.45 (2.15)	23.85 (10.49)	20.98 (8.86)	2.76 (2.39)	<0.001
	GT(s)	1.77 (0.50)	1.63 (0.475)	0.20 (0.19)	1.85 (0.69)	1.71 (0.635)	0.19 (0.20)	0.23
	TT(s)	2.28 (1.06)	2.09 (0.63)	0.16 (0.19)	2.30 (0.91)	2.01 (0.87)	0.31 (0.19)	<0.001
DTW	DTWT(s)	30.97 (14.85)	27.66 (12.40)	2.01 (3.76)	30.10 (9.84)	25.77 (8.36)	3.45 (2.66)	0.067
	GT(s)	1.86 (0.60)	1.75 (0.50)	0.17 (0.23)	1.76 (0.46)	1.64 (0.58)	0.125 (0.178)	0.29
	TT(s)	2.19 (1.16)	2.08 (0.92)	0.13 (0.24)	2.57 (0.89)	2.08 (0.86)	0.40 (0.273)	<0.001
POMA		20 (7)	20 (6)	1 (1.75)	19 (8)	20 (7)	2 (1)	<0.001
FAC		3 (1)	3 (1.5)		3 (1)	4 (1)		0.033

T0, assessment at baseline; T1, assessment after 2 weeks of treatment. Difference, the improvement value of the T1 indicator compared to T0. TUG, Timed up and Go test; TUGT, total time of TUG; GT, getting-up time of TUG/DTW; TT, turning time of TUG/DTW; DTW, Dual-Task Walking test; DTWT, total time of DTW; FAC, Functional Ambulation Category; POMA, Tinetti Performance Oriented Mobility Assessment. The numbers represent median (interquartile range). The *p* value was calculated using the Mann–Whitney U test on the improvement values (“Difference”) of the two groups (except for the FAC index) or the Wilcoxon rank sum test on the FAC at T1.

After 2 weeks of treatment, it was observed that rTMS plus conventional rehabilitation significantly outperformed conventional rehabilitation in improving TUG and POMA scores [2.76 (2.39) versus 1.45 (2.15), *p* < 0.001; 2 (1) versus 1 (1.75), *p* < 0.001]. Nonetheless, no comparable impact was detected in DTW [3.45 (2.66) versus 2.01 (3.76), *p* = 0.067] and neither was GT [0.19 (0.20) versus 0.20 (0.19), *p* = 0.23; 0.125 (0.178) versus 0.17 (0.23), *p* = 0.29]. Furthermore, the effectiveness of rTMS plus conventional rehabilitation in improving TT was greater than conventional rehabilitation in both TUG and DTW [0.31 (0.19) versus 0.16 (0.19), *p* < 0.001; 0.40 (0.273) versus 0.13 (0.24), *p* < 0.001]. The findings indicate that the combination of conventional rehabilitation training and rTMS plus conventional rehabilitation may enhance postural control, balance function, and anti-interference ability (specifically turn time improvement) for acute ischemic stroke patients.

After a 2-week intervention, the experimental group showed a notably more significant improvement in FAC compared to the control group [4 (1) versus 3 (1), *p* = 0.033]. This implies that rTMS plus conventional rehabilitation can enhance patients’ walking capabilities and decrease their reliance on external assistance.

### Secondary outcome measures (gait parameters)

In the single-task walking test, rTMS plus conventional rehabilitation resulted in significantly greater improvements in the following gait parameters (gait velocity, gait stride length, gait cadence, and gait cycle) than conventional rehabilitation [0.11 (0.085) versus 0.03 (0.049), *p* < 0.001; 0.06 (0.125) versus 0.03 (0.06), *p* < 0.001; 0.13 (0.11) versus 0.025 (0.095), *p* = 0.02; 0.048 (0.045) versus 0.0106 (0.0351), *p* < 0.001] (Table 3).

**Table 3:** Comparison of video-based gait analysis between two groups.

Variable		Control group (*n* = 36)			Experimental group (*n* = 37)			*p*
		T0	T1	Difference	T0	T1	Difference	
TUG (single-task)	Gait velocity (m/s)	0.61 (0.325)	0.64 (0.29)	0.03 (0.049)	0.60 (0.275)	0.76 (0.29)	0.11 (0.085)	<0.001
	Stride length (m)	0.83 (0.38)	0.85 (0.33)	0.03 (0.06)	0.72 (0.28)	0.83 (0.28)	0.06 (0.125)	<0.001
	Cadence (step/s)	1.58 (0.27)	1.58 (0.28)	0.025 (0.095)	1.62 (0.37)	1.72 (0.33)	0.13 (0.11)	0.02
	Cycle(s)	0.63 (0.11)	0.64 (0.11)	0.01 (0.04)	0.62 (0.14)	0.58 (0.12)	0.05 (0.05)	<0.001
DTW (dual-task)	Gait velocity (m/s)	0.45 (0.27)	0.51 (0.25)	0.03 (0.03)	0.52 (0.29)	0.61 (0.33)	0.08 (0.065)	0.002
	Stride length (m)	0.68 (0.35)1	0.73 (0.28)	0.03 (0.073)	0.71 (0.24)	0.77 (0.32)	0.045 (0.068)	0.12
	Cadence (step/s)	1.45 (0.42)	1.50 (0.50)	0.025 (0.07)	1.60 (0.36)	1.63 (0.35)	0.1 (0.12)	0.005
	Cycle(s)	0.69 (0.19)	0.67 (0.22)	0.0124 (0.0381)	0.63 (0.15)	0.61 (0.13)	0.036 (0.038)	0.038

T0, assessment at baseline; T1, assessment after 2 weeks of treatment. Difference, the improvement value of the T1 indicator compared to T0. *p* value was calculated according to the Wilcoxon rank-sum test.

In the dual-task walking test, rTMS plus conventional rehabilitation resulted in significantly greater improvements in gait velocity, gait cadence, and gait cycle [0.08 (0.065) versus 0.03 (0.03), *p* = 0.002; 0.1 (0.12) versus 0.025 (0.07), *p* = 0.005; 0.036 (0.038) versus 0.0124 (0.0381), *p* = 0.038], in comparison to conventional rehabilitation, excluding gait stride length [0.045 (0.068) versus 0.03 (0.073), *p* = 0.12].

The analysis of the gait parameters mentioned above suggests that rTMS plus conventional rehabilitation can improve walking ability in individuals who have had a stroke through various routes, indicating that it is not a straightforward enhancement.

### Secondary outcome measures (ADL, MMSE, MoCA, and NIHSS)

**Figure 3:**
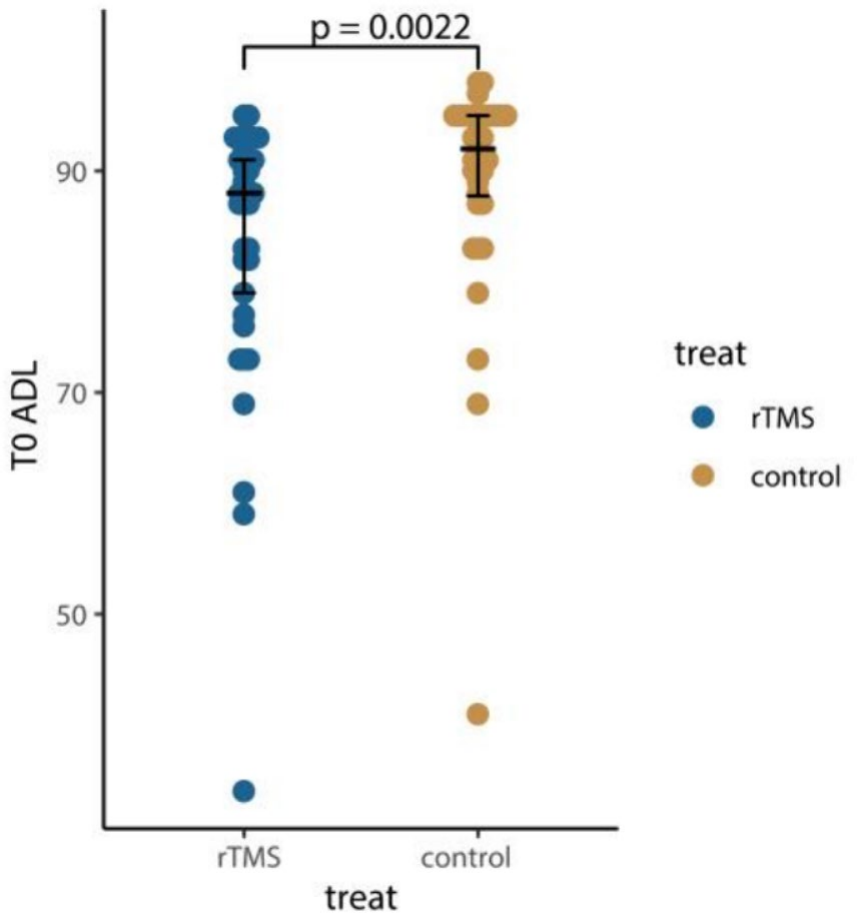
Comparison of ADL at baseline. T0, assessment at baseline; ADL, activities of daily living. *p* value was calculated according to the Wilcoxon rank-sum test.

Compared to conventional rehabilitation, rTMS plus conventional rehabilitation showed significant improvement in ADL (mesured by BI) and MoCA [3 (3) versus 0 (2.75), *p* < 0.001; 1 (1.5) versus 0 (1), *p* = 0.0043]. However, it is important to acknowledge that there was a significant statistical variance in ADL (mesured by BI) baseline measurements for the two groups [93 (8) versus 88 (9), *p* = 0.0022] (Figure 3; Table 4).

**Table 4:** Comparison of ADL, MMSE, MoCA, and NIHSS between two groups.

Variable	Control group (*n* = 36)			Experimental group (*n* = 37)			*p*
	T0	T1	Difference	T0	T1	Difference	
ADL	93 (8)	94 (5)	0 (2.75)	88 (9)	91 (7)	3 (3)	<0.001
MMSE	23.5 (3)	24 (5)	0 (1)	24 (7)	25 (6)	1 (1)	0.0058
MoCA	19 (4)	20 (5)	0 (1)	20 (7)	21 (7)	1 (1.5)	0.0043
NIHSS	1.5 (2)	1 (2)	0.5 (1)	2 (2)	1 (1)	1 (1)	0.707

T0, assessment at baseline; T1, assessment after 2 weeks of treatment. Difference, the improvement value of the T1 indicator compared to T0. ADL, activities of daily living; MMSE, Mini-Mental State Examination; MoCA, Montreal Cognitive Assessment; NIHSS, National Institutes of Health Stroke Scale. *p* value was calculated according to the Wilcoxon rank-sum test.

### Mediation effect analysis

The mediator was MoCA measured 2 weeks after the intervention. For each single mediator model, the intervention mediator effect, the mediator outcome effect, the total natural indirect effect (TNIE), the pure natural direct effect (PNDE), and the total effect (TE) were estimated, with the mediator’s proportion being the fraction of the TE explained by the TNIE.

**Figure 4:**
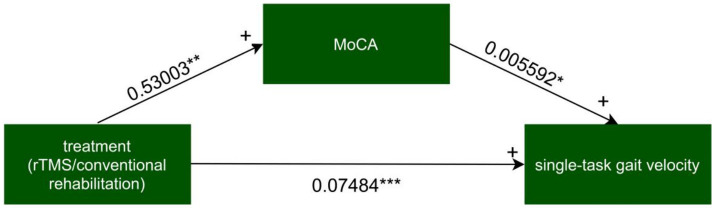
The mediated model of single-task gait velocity. rTMS, repetitive transcranial magnetic stimulation plus conventional rehabilitation.

**Figure 5:**
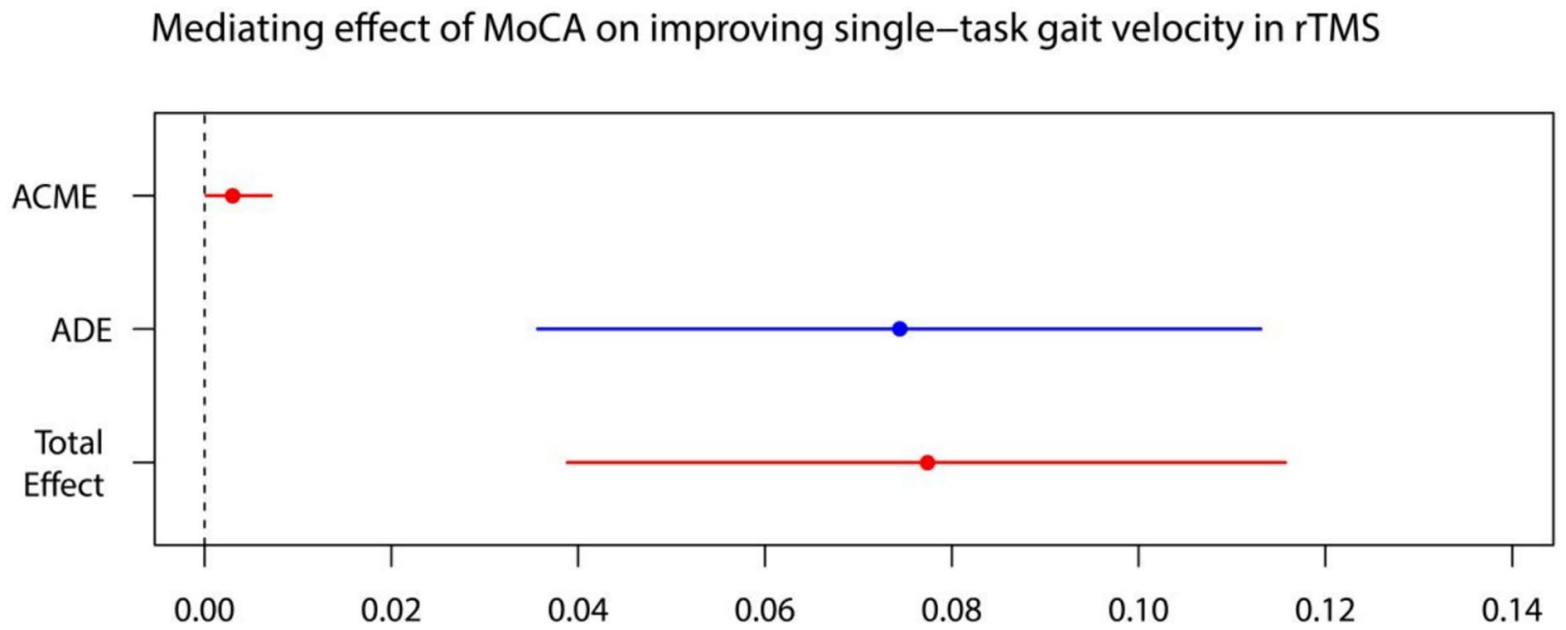
MoCA on single-task gait velocity. ADE, average direct effects; ACME, average casual mediation effects; rTMS, repetitive transcranial magnetic stimulation plus conventional rehabilitation.

**Figure 6:**
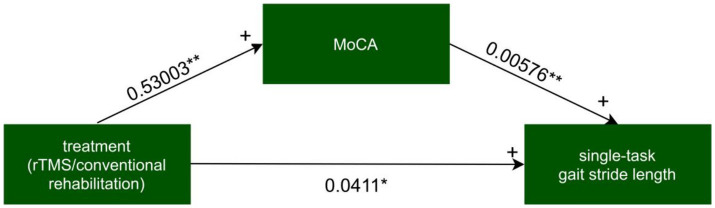
The mediated model of single-task gait stride length. rTMS, repetitive transcranial magnetic stimulation plus conventional rehabilitation.

**Figure 7:**
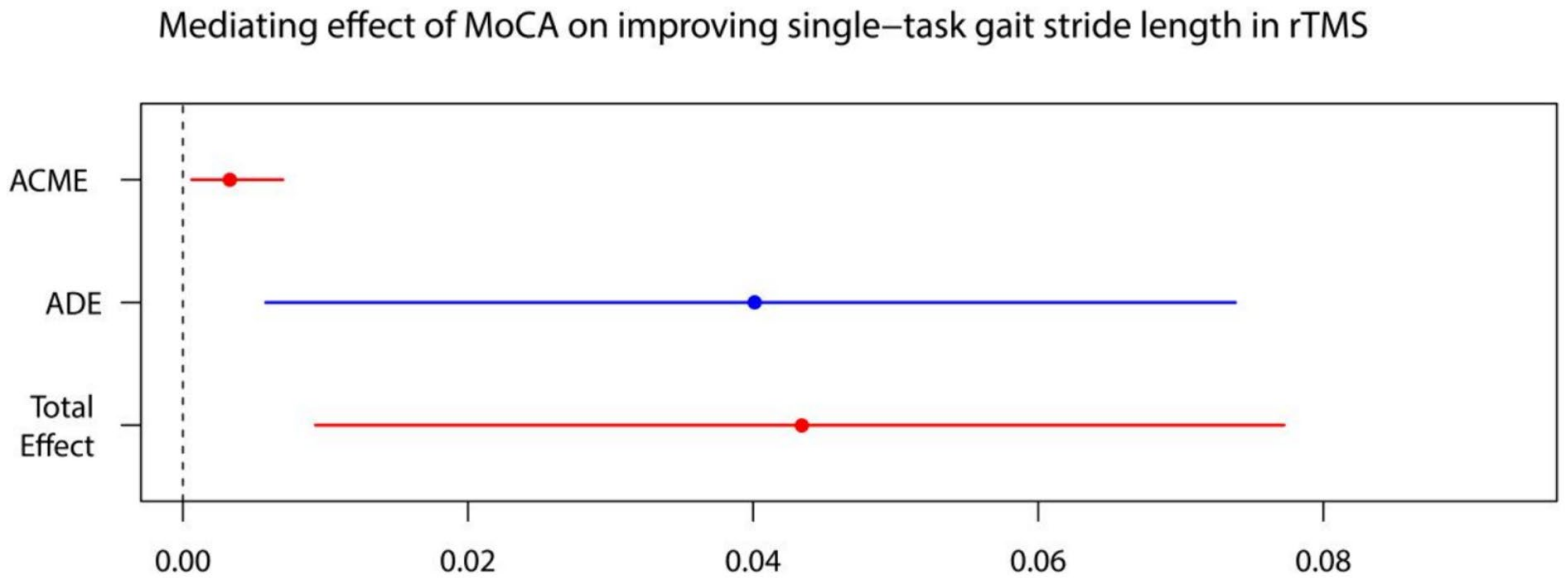
MoCA on single-task gait stride length. ADE, average direct effects; ACME, average casual mediation effects; rTMS, repetitive transcranial magnetic stimulation plus conventional rehabilitation.

For every independent mediated model, we fit two regression models: one mediated model and one outcome model. The mediated model utilized linear regression with the treatment measure as the independent variable, post-intervention MoCA as the dependent variable, and baseline MoCA as covariates. The outcome model for single-task gait velocity/single-task gait stride length was constructed with post-intervention MoCA as the independent variable, post-intervention single-task gait velocity/single-task gait stride length as the dependent variable, and treatment measures, and baseline value of single-task gait velocity/single-task gait stride length as covariates (Figures 4–7).

Causal mediation analysis revealed that alterations in cognitive function may partially underlie the improvements observed in single-task gait velocity and single-task gait stride length resulting from rTMS plus conventional rehabilitation. There was a significant indirect effect of cognitive function on single-task gait velocity and single-task gait stride length. The proportion of TE in single-task gait velocity and single-task gait stride length mediated by cognitive function was 0.036 and 0.073, respectively.

**Figure 8:**
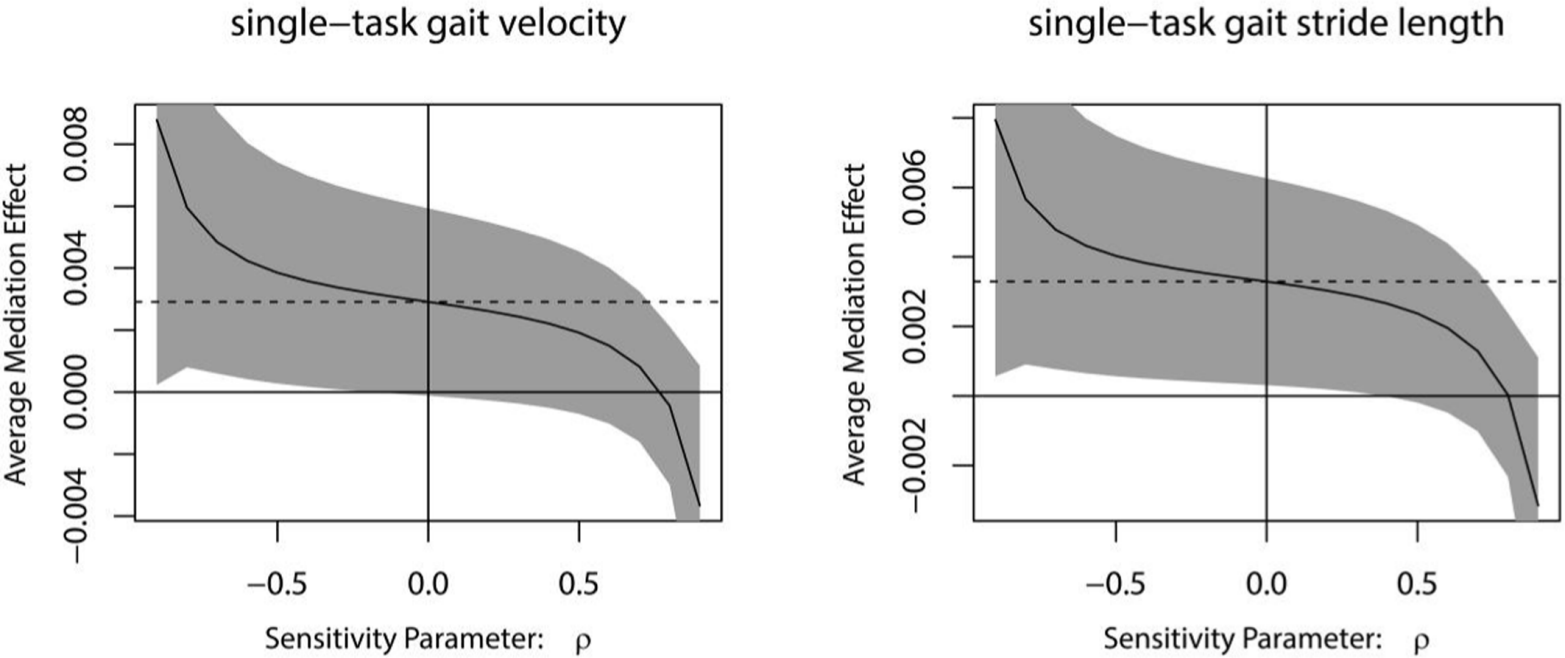
Sensitivity analysis.

The results of the sensitivity analyses indicated that the mediating impact of MoCA on rTMS plus conventional rehabilitation to enhance single-task gait velocity/single-task gait stride length becomes non-significant when rho is equal to 0.8/0.8 (Figure 8).

### Long-term effects of rTMS plus conventional rehabilitation on postural control

**Figure 9:**
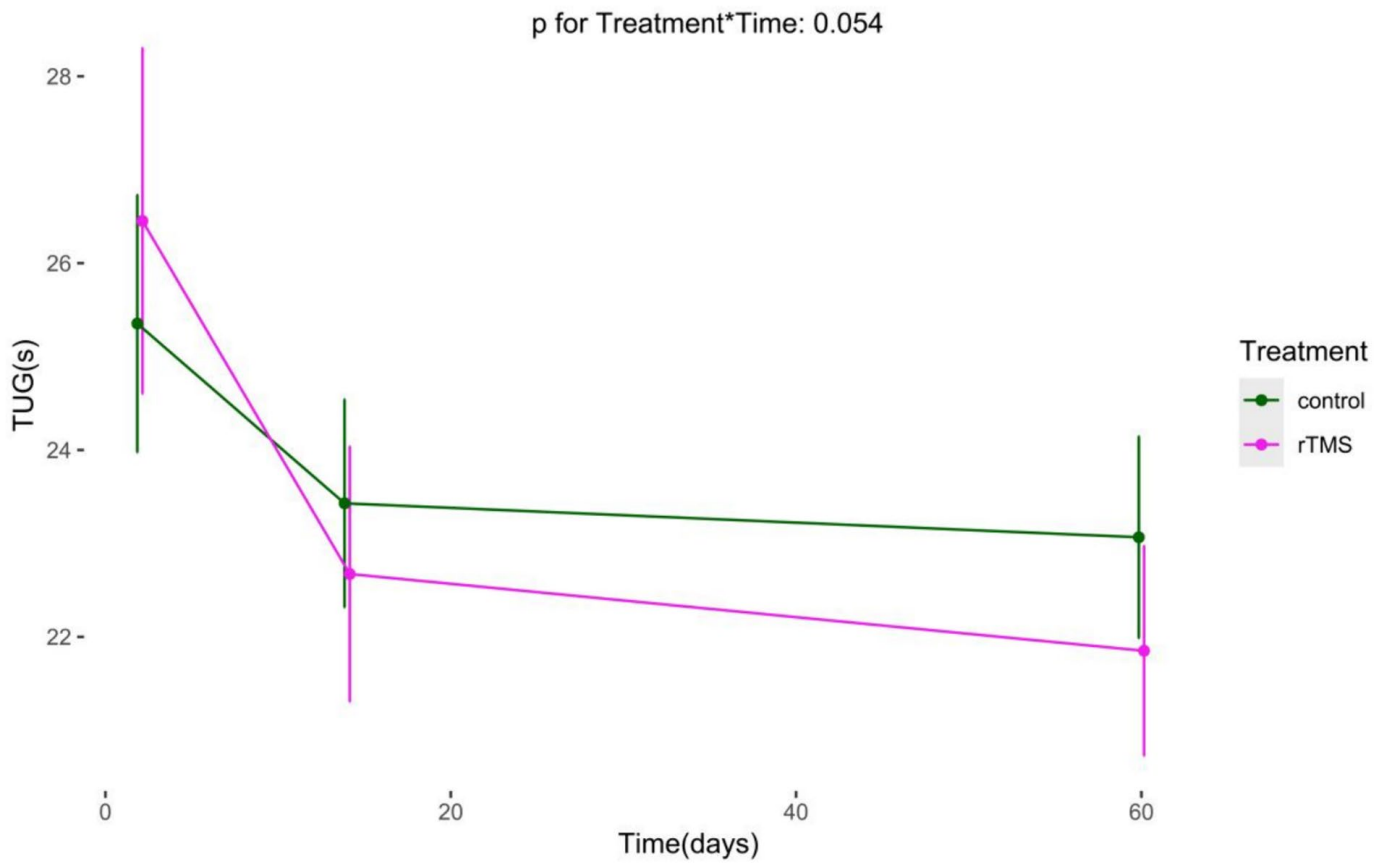
The Long-term changes in TUG. rTMS, repetitive transcranial magnetic stimulation plus conventional rehabilitation.

**Figure 10:**
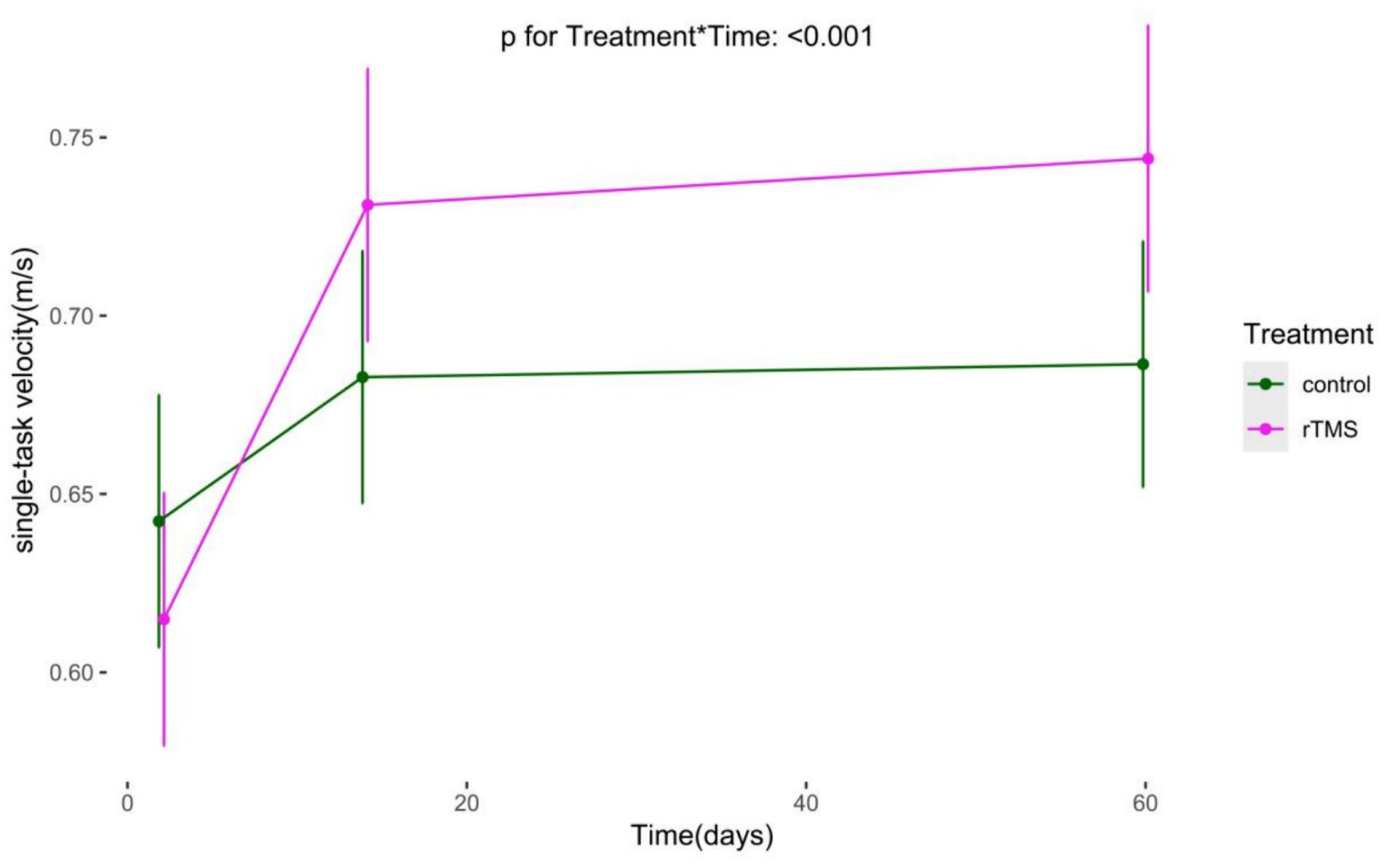
The Long-term changes in single-task gait velocity. rTMS, repetitive transcranial magnetic stimulation plus conventional rehabilitation.

We conducted an analysis on TUG and single-task gait speed, using generalized estimating equations with robust standard errors and exchangeable job correlation matrices. Our findings reveal marked improvements in both groups’ TUG and single-task gait velocity over time. Additionally, no statistically significant difference in the trend of TUG over time was observed in the experimental group compared to the control group (*p* for Treatment*Time = 0.054). The experimental group demonstrated a significantly superior trend in single-task gait velocity increase over time compared to the control group (*p* for Treatment*Time < 0.001) (Figures 9, 10).

### Effects of rTMS plus conventional rehabilitation on gait recovery

**Figure 11:**
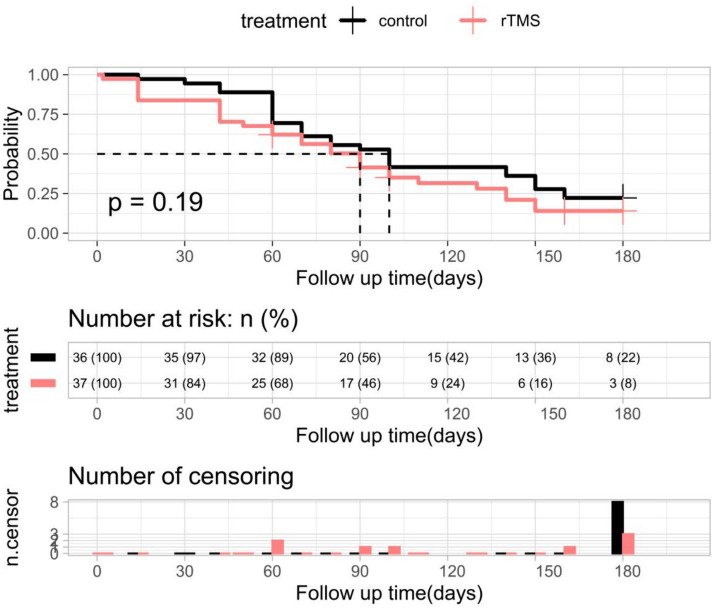
K-M curves and log-rank tests for independent walking recovery in the two groups of patients. rTMS, repetitive transcranial magnetic stimulation plus conventional rehabilitation.

The K-M curves of independent walking recovery in the two groups are shown in Figure 11, and the results of the log-rank test showed that the median time for gait recovery was 90 days for patients in the experimental group and 100 days for those in the control group. However, there was no statistically significant difference in the trends of the two curves (*p* = 0.19).

Multifactorial Cox regression analyses showed that rTMS plus conventional rehabilitation had a significant impact on facilitating independent walking recovery in patients when compared to conventional rehabilitation (Table 5). Age, gender, initial NIHSS score, and Brunnstrom staging influenced gait recovery (Table 5).

**Table 5:** Multifactorial Cox regression analysis influencing independent walking recovery.

Variables	aHR	95% CI	*p*
Treatment (rTMS plus conventional rehabilitation vs. conventional rehabilitation)	2.76	1.50–5.09	0.001
Age (per year)	0.96	0.94–0.99	0.010
Gender (Female vs. Male)	0.43	0.21–0.86	0.011
Brunnstrom stage (per additional stage)	1.59	1.00–2.51	0.048
Initial NIHSS score (per point)	0.79	0.63–0.98	0.032

NIHSS, US National Institutes of Health Stroke; aHR, adjusted hazard ratio; CI, confidence interval.

### Safety

No significant adverse events were identified during the study.

## Discussion

The objective of this project was to evaluate the efficacy of high-frequency (10 Hz) rTMS on the M1 area of the lesion side and the left DLPFC area plus conventional rehabilitation in enhancing postural control ability in patients with cerebral infarction in the acute phase (< 1 month). The analyzed results indicate that after two weeks of intervention, rTMS plus conventional rehabilitation led to significant improvements in TUG, TT, gait velocity, gait cadence, gait cycle, stride width, single-task gait stride length, FAC, and POMA, compared to traditional rehabilitation therapy. The mediation analyses indicated that the improvement of MoCA may have played a mediating role in the improvement of single-task gait velocity and gait stride length by rTMS plus conventional rehabilitation. The GEE analyses revealed a significant long-term effect of rTMS plus conventional rehabilitation on single-task gait velocity, but not on TUG. The cox regression analysis indicated that rTMS plus conventional rehabilitation can enhance the recovery of walking ability. A parallel randomized controlled trial design was adopted to prevent interference from the natural recovery of the disease and to more accurately observe the positive rehabilitation effect of rTMS.

Normal areas surrounding the lesion site or the healthy cerebral hemisphere may contribute to the recovery of lower limb function after cerebral infarction (41). The ability of the brain to adapt to changes in the environment is known as neuroplasticity. For patients with cerebral infarction, part of the neural function is lost due to necrosis and apoptosis of nerve cells, and the remaining synaptic network can be further activated, so that the remaining neural resources can be fully utilized and redistributed, thus compensating for the missing neural function (42). Under normal conditions, the two cerebral hemispheres regulate each other’s excitability through the corpus callosum connection, maintaining a balance between them (43). However, in patients with cerebral infarction, there is an imbalance of inhibition between the hemispheres (44), leading to impaired excitability of the motor cortex in the affected hemisphere and affecting limb movement (45). The mechanism of action of rTMS to improve lower limb motor function in patients with cerebral infarction is to intervene in cortical reorganization through neuromodulation of rTMS and thereby improve motor function. High-frequency rTMS increases cortical excitability on the lesion side, while low-frequency rTMS decreases cortical excitability on the healthy side, resulting in a balance of excitability in both cerebral hemispheres to improve motor function in patients with cerebral infarction (46–48). Previous studies by Sasaki have examined the safety and efficacy of high-frequency rTMS stimulation of the M1 region applied to leg motor function in patients in the early stages of stroke (49). Accordingly, the M1 region rTMS stimulation protocol employed in this study was based on the Sasaki protocol.

Among the primary outcome indicators in our study, rTMS plus conventional rehabilitation improved patients’ postural control (TUG, Tinetti). Kang’s meta-analysis showed that rTMS had a positive effect on improving postural control (BBS, PASS) (26). However, the results of several clinical studies and meta-analyses have shown that rTMS had no significant improvement in postural control (TUG) (50, 51). We speculate that it may be based on the following reasons. First, the TUG test assesses various aspects of lower limb muscle strength, balance, and mobility (52). Our study used a modified version of the conventional TUG, with a longer distance of 5 meters instead of the usual 3 meters. This modification may be more effective in detecting small differences in treatment effects. Additionally, the percentage of straight-walking time increased in the 5-meter TUG and the improvement of TUG in the test group may be primarily attributed to the enhancement of walking speed. Second, our study had a shorter observation period, focusing on changes in TUG during the acute phase (within 2 weeks), while most other studies observed the therapeutic effect over a longer period of time (50, 51). The duration of observation may have contributed to the difference in the study results. Our 2-month follow-up results confirmed this. The GEE analysis showed that the trend of TUG change from pre-intervention to 2 months was not significantly different between the two patient groups. This suggests that rTMS improves postural control in the acute phase (< 2 week) but does not make a difference in the subacute phase (14–60 days). It may simply shorten the patient’s rehabilitation time for postural control. Our multifactorial Cox regression analysis of patients’ independent walking recovery also showed that rTMS could shorten the process of independent walking recovery in stroke patients.

Regarding improvement in Functional Ambulation Category (FAC), we analyzed the differences in TUG, stride speed, and stride length among the different categories of FAC in the included population. The general trend was that higher FAC scores were associated with shorter TUG times, faster stride speeds, and longer stride lengths. Previous studies by Jan have shown a strong relationshi*p* between the FAC category and the Rivermead Mobility Index (RMI), gait velocity, and gait stride length (33). Therefore, the improvement of the FAC may be closely related to the improvement of the TUG test and gait parameters. Walking is a multifaceted process that involves various parameters, such as gait speed, gait width, gait cadence, gait stride length, and gait cycle. The modulation of gait parameters is regulated by multiple neural systems, including cortical, subcortical, and spinal networks (51). Functional magnetic resonance studies have shown that various brain areas, such as the primary sensorimotor area, the primary motor area, the supplementary motor area, the basal ganglia, and the cerebellar vermis, are activated in response to increased blood flow during normal walking. This study suggests that rTMS plus conventional rehabilitation improves not only lower limb motor function in stroke patients through direct site of action but also rehabilitation efficacy through connections between different stimulation sites and brain regions (42). The integration of the lesion-side M1 region into the motor network structure may play a key role in improving motor function in stroke patients through rTMS (42).

Regarding the effect of rTMS plus conventional rehabilitation on waling speed in stroke patients, it is consistent with Li′s meta-analysis (51). Our GEE analyses demonstrated the long-term effects of rTMS plus conventional rehabilitation on walking speed, which may be attributed to the physiological effects of TMS signals, such as the stimulation of gene expression and enzyme production in the body (53). However, Chieffo found that 20 Hz high-frequency rTMS did not increase walking speed in patients with chronic stroke, although it significantly improved lower limb motor function (54). In contrast, our study focused on patients with acute-phase cerebral infarction, where stroke-induced changes in neuroplasticity are crucial for motor function recovery. The relationship between the improvement in walking speed and the plasticity of neurological function is significant. This is because the plasticity period typically lasts for 1–3 months, after which the plasticity of neurological function decreases (55). Therefore, early rTMS rehabilitation is crucial for patients with cerebral infarction.

The mediation analyses indicated that cognitive function improvements (MoCA) mediate the enhancement of single-task gait velocity and gait stride length by rTMS (M1 + DLPFC) plus conventional rehabilitation. This suggests that stimulating different sites may improve the connectivity between these areas and thus enhance rehabilitation efficacy (42). Previous literature has demonstrated a significant correlation between motor and cognition in the elderly population. The two have a longitudinal effect on each other (56), with improvements in cognitive domains, such as executive function, attention, and visuospatial function, being associated with increases in walking speed (57). The left-lateral DLPFC area is crucial in regulating higher cognitive functions, such as memory, attention, and executive functioning. Yin demonstrated that high-frequency rTMS applied to this area significantly improved the executive functioning of patients with post-stroke cognitive impairment (58). Executive functions are cognitive processes that individuals use to control and regulate basic cognitive processes, producing orderly, goal-directed behaviors (59). Executive function is closely related to the completion of daily activities, including motion. Accordingly, in the present study, we employed Yin’s rTMS stimulation protocol with the objective of enhancing the patient’s executive function by stimulating the left DLPFC area (58), which, in turn, facilitated the improvement of postural control. Nevertheless, rTMS alone has a limited role in the rehabilitation of stroke patients. It only provides a temporary window of rehabilitation. Since rTMS only enhances nerve plasticity and prolongs the effectiveness of rehabilitation training, its true benefits can only be realized when used in conjunction with corresponding neurological training during this period (42).

In the multifactorial Cox regression model, rTMS plus conventional rehabilitation has been demonstrated to facilitate the functional recovery of independent gait in patients who have experienced a stroke. However, it is important to note that a number of other factors, including age, sex, Brunnstrom stage, NIHSS score, also exert a significant influence on the recovery of gait in these patients. Previous studies have demonstrated that advanced age is a predictor of diminished functional mobility in stroke patients (50, 60). Patients who have experienced more severe strokes tend to exhibit poorer functional mobility during the early stages of stroke onset. When this is coupled with advanced age, it may result in prolonged functional mobility recovery (61). A number of studies have investigated sex differences in functional outcomes following stroke. These studies have consistently demonstrated that women tend to exhibit poorer functional outcomes than men (62–65). Consequently, it is imperative to consider the incorporation of rTMS into the rehabilitation programs of women, older patients and stroke patients with higher NIHSS scores. An accurate assessment of the Brunnstrom stage at the same time allows for targeted rehabilitation and functional prediction.

## Conclusion

Repetitive transcranial magnetic stimulation (rTMS) plus conventional rehabilitation is somewhat more effective than conventional rehabilitation alone in improving postural control in patients with mild hemiparesis following acute ischemic stroke. The favorable therapeutic effect of rTMS on postural control may be partly mediated by improvements in cognitive function.

## Limitations

There are limitations to this study. One major limitation is that the participants included had experienced acute cerebral infarction no more than a week prior. Therefore, the current effect of rTMS on rehabilitation cannot be distinguished from the effect of natural recovery from the disease course in patients with cerebral infarction. However, we selected both control and test group patients who underwent necessary routine rehabilitation to minimize bias in the results due to natural recovery. Another limitation is that these results did not incorporate ≥3 months of follow-up assessment data as an outcome measure. Therefore, attempts to extrapolate the current findings to a long-term clinical prognosis should be interpreted with caution. Nonetheless, a longer follow-up assessment was performed, except that a different indicator than the preexisting outcome indicator was used. In future studies, it may be worth considering the combination of rTMS and motor-cognitive dual-task training to investigate whether cognitive training can enhance the efficacy of rTMS in improving exercise. Additionally, conducting long-term follow-up could help clarify the interaction and changes between cognition and exercise in patients with cerebral infarction.

## Data availability statement

The raw data supporting the conclusions of this article will be made available by the authors, without undue reservation.

## Ethics statement

The study procedure was reviewed and approved by the Ethics Committee of the Shanghai Tenth People’s Hospital (SHSY-IEC-BG/05.08/05.0), and was registered in the Chinese Clinical Trial Registry (No. ChiCTR1900026225). All participating patients were duly informed of the study procedures and its safety and signed an informed consent form. The study was conducted in compliance with the Declaration of Helsinki principles.

## Author contributions

JM: Data curation, Methodology, Visualization, Writing – original draft. SQ: Supervision, Validation, Writing – review & editing. NM: Visualization, Writing – review & editing. LZ: Investigation, Writing – review & editing. LX: Writing – review & editing. XL: Conceptualization, Data curation, Funding acquisition, Resources, Writing – review & editing. GM: Data curation, Funding acquisition, Methodology, Project administration, Resources, Writing – review & editing.
